# The Role of Interstitial Matrix and the Lymphatic System in Gastrointestinal Lipid and Lipoprotein Metabolism

**DOI:** 10.3389/fphys.2020.00004

**Published:** 2020-01-22

**Authors:** Anna Zhou, Jie Qu, Min Liu, Patrick Tso

**Affiliations:** Department of Pathology and Laboratory Medicine, University of Cincinnati Reading Campus, Cincinnati, OH, United States

**Keywords:** lymphatic circulation, chylomicron, mucosal mast cell, microbiome, lipids, gastrointestinal tract, enterocytes, intestinal absorption

## Abstract

This review emphasizes the events that take place after the chylomicrons are secreted by the enterocytes through exocytosis. First, we will discuss the journey of how chylomicrons cross the basement membrane to enter the lamina propria. Then the chylomicrons have to travel across the lamina propria before they can enter the lacteals. To understand the factors affecting the trafficking of chylomicron particles across the lamina propria, it is important to understand the composition and properties of the lamina propria. With different degree of hydration, the pores of the lamina propria (sponge) changes. The greater the hydration, the greater the pore size and thus the easier the diffusion of the chylomicron particles across the lamina propria to enter the lacteals. The mechanism of the entry of lacteals is discussed in considerable details. We and others have demonstrated that intestinal fat absorption, but not the absorption of protein or carbohydrates, activates the intestinal mucosal mast cells to release many products including mucosal mast cell protease II in the rat. The activation of intestinal mucosal mast cells by fat absorption involves the process of chylomicron formation since the absorption of both medium and short-chain fatty acids do not activate the mast cells. Fat absorption has been associated with increased intestinal permeability. We hypothesize that there is a link between fat absorption, activation of mucosal mast cells, and the leaky gut phenomenon (increased intestinal permeability). Microbiome may also be involved in this chain of events associated with fat absorption. This review is presented in sequence under the following headings: (1) Introduction; (2) Structure and properties of the gut epithelial basement membrane; (3) Composition and physical properties of the interstitial matrix of the lamina propria; (4) The movement of chylomicrons across the interstitial matrix of the lamina propria and importance of the hydration of the interstitial matrix of the lamina propria and the movement of chylomicrons; (5) Entry of the chylomicrons into the intestinal lacteals; (6) Activation of mucosal mast cells by fat absorption and the metabolic consequences; and (7) Link between chylomicron transport, mucosal mast cell activation, leaky gut, and the microbiome.

## Introduction

The absorption and transport of lipids by the gastrointestinal tract involve the uptake of digested lipids by the enterocytes and the formation and secretion of chylomicrons. These different steps of intestinal fat absorption have been so ably reviewed by a number of reviews over the past few years (Wang et al., 2013; Dash et al., 2015; Levy, 2015; D’Aquila et al., 2016; Mansbach and Siddiqi, 2016; Auclair et al., 2018; Xiao et al., 2019). In most textbooks and review articles, fat absorption is described as involving the packaging of the absorbed lipids into chylomicrons, the exit of the chylomicrons from the enterocytes through exocytosis, and the subsequent entry of the chylomicrons into the lymphatics of the gastrointestinal tract. Electron microscopic studies have contributed significantly to our understanding of the formation and secretion of chylomicrons and the review by Sabesin and Frase (1977) remain one of the best articles describing this process. The role of the lymphatic system in fat absorption has been recognized for centuries. As early as the 1600s, Italian physician Gaspare Aselli demonstrated a post-prandial rise in the concentration of particulate fat in the mesenteric lymph of a dog fed shortly before its death (Yoffey and Courtice, 1970; Barrowman, 1978). A major advancement in our understanding of the transport of fat by the lymphatic system was enabled by the conscious lymph fistula rat and mouse studies (Hayashi et al., 1990; Kohan et al., 2012; Ko et al., 2019).

In fact, many events are involved from the secretion of the chylomicrons by the enterocytes to the subsequently transport of these triglyceride-rich lipoproteins by the lymphatic system. One of the goals of this review is to cover those events that occur from the secretion of chylomicrons *via* exocytosis by the enterocytes to the final entry of the chylomicrons into the lymph lacteals of the intestinal villi. After exiting from the enterocytes, the chylomicrons accumulate in the intercellular space. The basement membrane with which the enterocytes are attached to offers considerable resistance for the passage of chylomicrons from the intercellular space into the lamina propria. We will discuss how we think the chylomicrons cross the basement membrane to enter into the lamina propria. We will also discuss the properties of the lamina propria and the factors (e.g., hydration of the lamina propria) influencing the diffusion of the chylomicrons across the lamina propria and how the chylomicrons subsequently enter the lacteals located in the central core of the villus. The lacteals transporting the chylomicrons drain initially into the intestinal lymph duct, then the thoracic duct, and finally empty into the left subclavian vein.

Interest in the lymphatic system increased dramatically recently in its role in lipid metabolism and gastrointestinal function. We and others have demonstrated that in addition to chylomicrons, the lymphatics of the gastrointestinal tract also carry molecules secreted by the mucosal mast cells [mucosal mast cell protease II in the rat (Ji et al., 2011; Sato et al., 2016)] and mucosal mast cell protease I in the mouse (Miller and Pemberton, 2002) when these cells are activated during fat absorption. This finding is not surprising given the close association between the lymphatic vessels and the mucosal mast cells that has been so elegantly demonstrated by Chatterjee and Gashev (2012). Our better understanding of lymphatic transport of particles ranging in size from large chylomicrons to small mast cell activation products and incretin hormones (GLP-1 and GIP) clearly emphasizes the importance of the intestinal lymphatic system in the transport of many important molecules during health and the diseased state. With the recent surge in interest in the intestinal microbiome, we anticipate a better understanding of the molecules derived from the microbiome carried by the lymphatic system. We may also gain insight into the importance of the microbiome in the well-being of the gastrointestinal tract as well as the origin and development of conditions associated with the leaky gut and gut inflammation.

## Structure and Properties of the Gut Epithelial Basement Membrane

During active lipid absorption, monoglycerides and fatty acids produced from the digestion of triglycerides are taken up by enterocytes. Here, they are re-esterified to produce triglycerides and are packaged into chylomicrons for export into the lymphatic system. For a more comprehensive discussion of these processes, readers are referred to the following excellent treatises on the subject (Levy, 1992, 2015; Abumrad and Davidson, 2012; Wang et al., 2013; Hussain, 2014; Dash et al., 2015; D’Aquila et al., 2016; Mansbach and Siddiqi, 2016; Auclair et al., 2018; Xiao et al., 2019).

Basement membranes are present in every tissue of the human body and are found either as enveloping cell coats or as sheets underlying cell layers. The gut basement membrane is a specialized structure composed of extracellular matrix components that supports and separates the epithelium from the underlying lamina propria made up of connective tissue (Paulsson, 1992; Timpl, 1996). The primary constituents of most basement membranes include collagens (predominantly collagen IV), laminins, proteoglycans, calcium-binding proteins, as well as other structural or adhesion proteins (Timpl, 1996). Basement membranes are formed by a process of self-assembly (Paulsson, 1992). Initiation of basement membrane assembly in a tissue requires the synthesis of *α*, *β*, and *γ* laminin subunits and the formation of laminin heterotrimers (Yurchenco et al., 1997). Secreted laminins anchor to the cell surface through several receptors (Oh et al., 1997; Edwards et al., 1998; James et al., 2000) and self-assemble into a polymeric network (Yurchenco et al., 1985). This lattice serves as scaffolding for further basement membrane assembly, including the addition of a matrix polymer of collagen IV molecules (Pöschl et al., 2004). The compact network of overlaid laminin and collagen lattices creates a dense meshwork with pores ranging in size from ~10 to 130 nm (depending on the tissue involved) that allow the basement membrane to function as a selective barrier (Yurchenco and Ruben, 1987; Yurchenco et al., 1992; Abrams et al., 2003).

**Figure 1:**
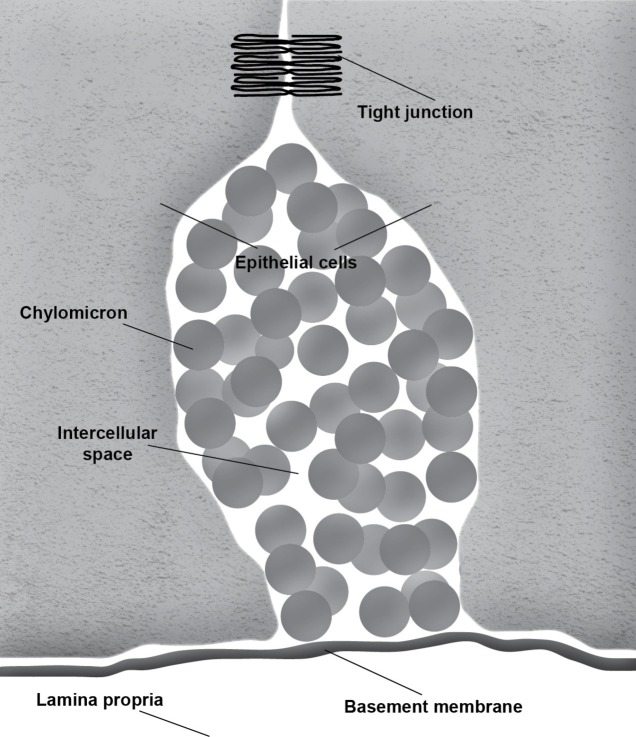
Accumulation of chylomicrons causes distension of the intercellular space. The intact basement membrane serves as a barrier preventing the chylomicrons from entering the underlying connective tissue of the lamina propria.

**Figure 2:**
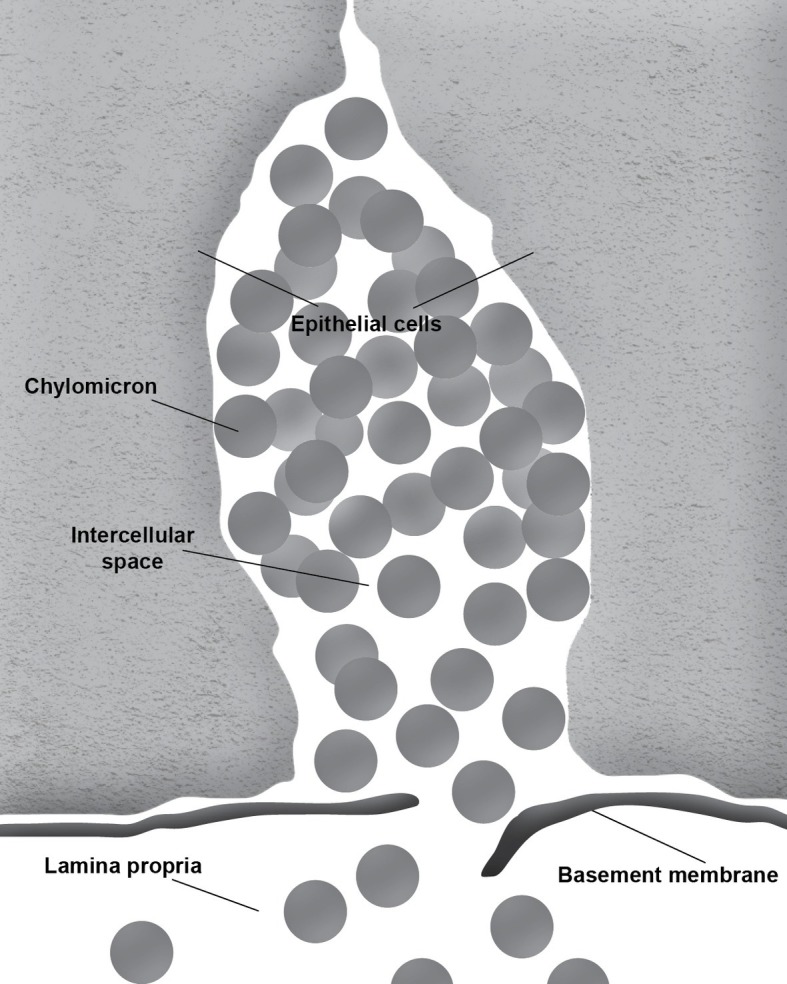
Chylomicrons enter the lamina propria through a break in the basement membrane.

During the absorption of fat, the gut basement membrane acts as a temporary barrier preventing chylomicrons, particles empirically defined as having a diameter equal to or larger than 800 Å, from entering the lamina propria (Figure 1). However, occasional discontinuities in the basement membrane allow the passage of the chylomicrons from the intercellular space to the lamina propria (Figure 2; Rubin, 1966; Tytgat et al., 1971; Sabesin and Frase, 1977). It is not uncommon to see the intercellular space between the gut epithelial cells full of chylomicrons during active fat absorption and in some images; the basement membrane appears to be broken. Thus, it has also been proposed that the basement membrane would be stretched under these conditions, causing breaks that would facilitate the transport of chylomicrons from the intercellular space to the lamina propria (Tso and Balint, 1986). However, this notion has not been tested experimentally. Kvietys et al. (1991) have demonstrated mucosal injury associated with intestinal fat absorption, but not with the absorption of the other macronutrients, which lends support to the notion that fat absorption is injurious to the integrity of the intestinal tract.

During fat absorption, the accumulation of chylomicrons in the intercellular space undoubtedly puts physical stress to the tight junctions. Furthermore, passage through breaks in the intestinal basement membrane is likely non-specific, which could allow molecules other than chylomicrons to enter the lamina propria. It is conceivable that this mode of entry may play a role in increasing intestinal permeability and the development of leaky gut. Thus, it is important for us to gain a better understanding of this process.

## Composition and Properties of the Interstitial Matrix of the Lamina Propria

The lamina propria of the small intestine is made up of connective tissue that occupies the central cone of the intestinal villus and offers physical support for the villus structure. Many cell types are present in the lamina propria, including mast cells, plasma cells, lymphocytes, eosinophils, neuronal cells, and fibroblasts. It also contains blood and lymph vessels, unmyelinated nerve fibers, and smooth muscle cells (Tso and Balint, 1986). The interstitial matrix of the lamina propria consists of a fluid phase that is distributed within fibrillar and non-fibrillar macromolecules. The primary fibrillary components include elastin and collagens, while non-fibrillar components include glycosaminoglycans, the major one being hyaluronic acid. The glycosaminoglycans trap water, which allows them to regulate the hydration of the interstitial matrix (Kvietys and Granger, 2010). The extensive crosslinking of the collagens and glycosaminoglycans results in a gel-like matrix within the lamina propria that behaves like a mesh and excludes large molecules from the fluid phase (Kvietys and Granger, 2010).

The interstitial volume and hydration are carefully maintained by the coordinated efforts of the blood and lymph vessels as well as fluid absorption from the intestinal lumen. During the resting in between meals, the balance of hydrostatic and oncotic forces governing trans-capillary fluid exchange, as described by the Starling principle, favors net filtration from the blood to the interstitium. This is balanced by an equal rate of fluid removal by the lymphatic vessels. During fluid absorption, alterations in the Starling forces allow the capillaries and lymphatics to remove the excess fluid in the interstitium and maintain the interstitial volume.

## The Movement of Chylomicrons Across the Interstitial Matrix of the Lamina Propria and the Importance of the Hydration of the Matrix

The properties of the lamina propria interstitial matrix play an important role in regulating chylomicron transport to the lymph lacteal centrally located within the intestinal villus. Once chylomicrons are transported across the epithelial basement membrane, they must then travel ≈50 μm through the interstitium before being taken up into the lacteal (Granger, 1981). Fluid absorption usually increases with the absorption of lipids and other nutrients, which subsequently increases the hydration of the interstitial matrix of the lamina propria. Ji et al. demonstrated that mucosal mast cells (MMC) are activated during fat absorption and the degranulation of the mast cells consequently releases molecules such as histamine (Ji et al., 2012). Histamine increases the permeability of the intestinal capillaries, thus dramatically increasing the filtration of molecules and fluid into the interstitial space of the villus. Evidence from previous studies (Shepherd and Simmonds, 1959; Baraona and Lieber, 1975; Granger, 1981; Granger et al., 1984; Tso et al., 1985) has suggested that the hydration of the interstitial matrix facilitates the transport of chylomicrons across the lamina propria. Two possible mechanisms have been proposed for this phenomenon.

First, hydration increases the permeability of the interstitial matrix. Under resting, non-absorptive conditions, the matrix has an average pore size of 250 Å, allowing it to exclude large molecules from the fluid phase. Thus, during the non-absorptive phase, the relatively small pore size of the interstitial matrix offers significant resistance to the diffusion of chylomicrons (particles empirically defined as having a diameter equal to or larger than 800 Å) through the intercellular space. During fluid absorption, the volume of the interstitium increases, resulting in the expansion and disentanglement of the matrix components. Under these conditions, the average porosity increases to 1,000 Å (Granger, 1981; Tso et al., 1985) which likely facilitates the movement of chylomicrons across the interstitial space to the central lacteals.

It has also been proposed that chylomicron transport could be facilitated by the convective fluid movement of lymph associated with fluid absorption. During lipid absorption, the subsequent increase in fluid uptake results in an increase in interstitial fluid formation and lymph flow. Previous studies have suggested that the rate of lymph formation has a major effect on chylomicron transport (Shepherd and Simmonds, 1959; Baraona and Lieber, 1975; Tso et al., 1985). Tso et al. studied the effect of lymph flow on chylomicron appearance time (time between infusion of radioactive fatty acid into the intestinal lumen and the appearance of radioactive lipid as chylomicrons in the intestinal lacteal). They reported that in a rat model, chylomicron appearance time was inversely proportional to lymph flow rate when the flow rate was <40 μl/min. When the flow rate exceeded 40 μl/min, chylomicron appearance rate plateaued at a minimal value of 13.6 min. This likely represented the minimum time necessary for the re-esterification of absorbed fatty acids and monoglycerides, chylomicron formation, and discharge into the lacteals (Tso et al., 1985).

It is still unclear if the increase in chylomicron transport associated with fluid absorption is a result of increased permeability of the interstitial matrix or the convective fluid movement. However, in both cases, it is apparent that hydration of the interstitial matrix plays an important role in the movement of chylomicrons across the lamina propria.

## Entry of the Chylomicrons Into the Lacteals

**Figure 3:**
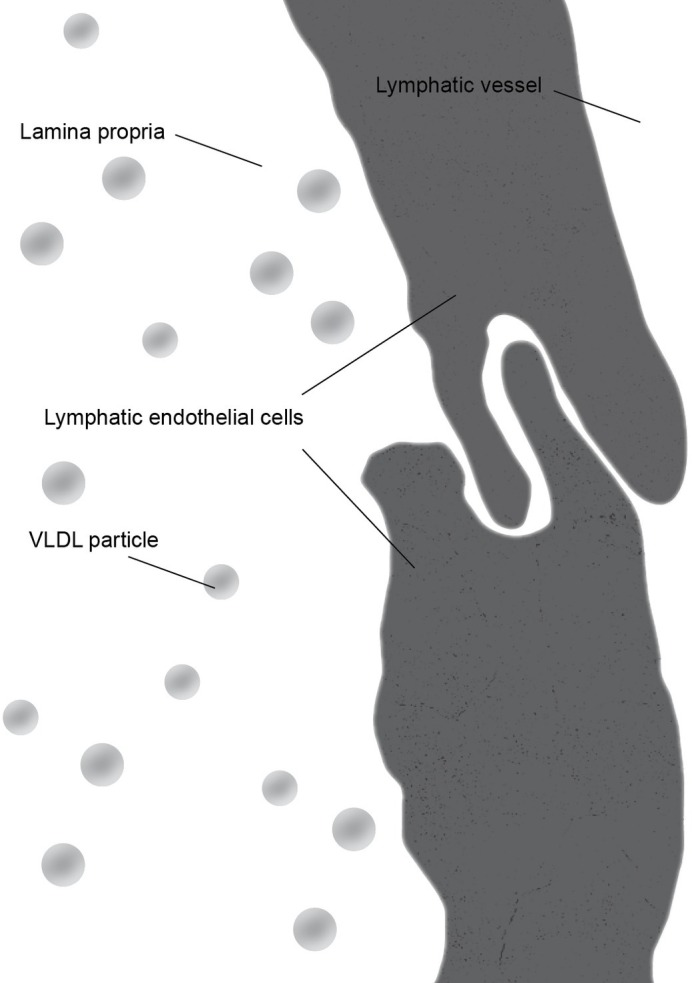
During the fasting state, junctions between lymphatic endothelial cells form a continuous cellular lining of the central lacteal of an intestinal villus. The primary lipoprotein particles present in the lamina propria are very low density lipoprotein (VLDL) particles which are significantly smaller than chylomicrons.

**Figure 4:**
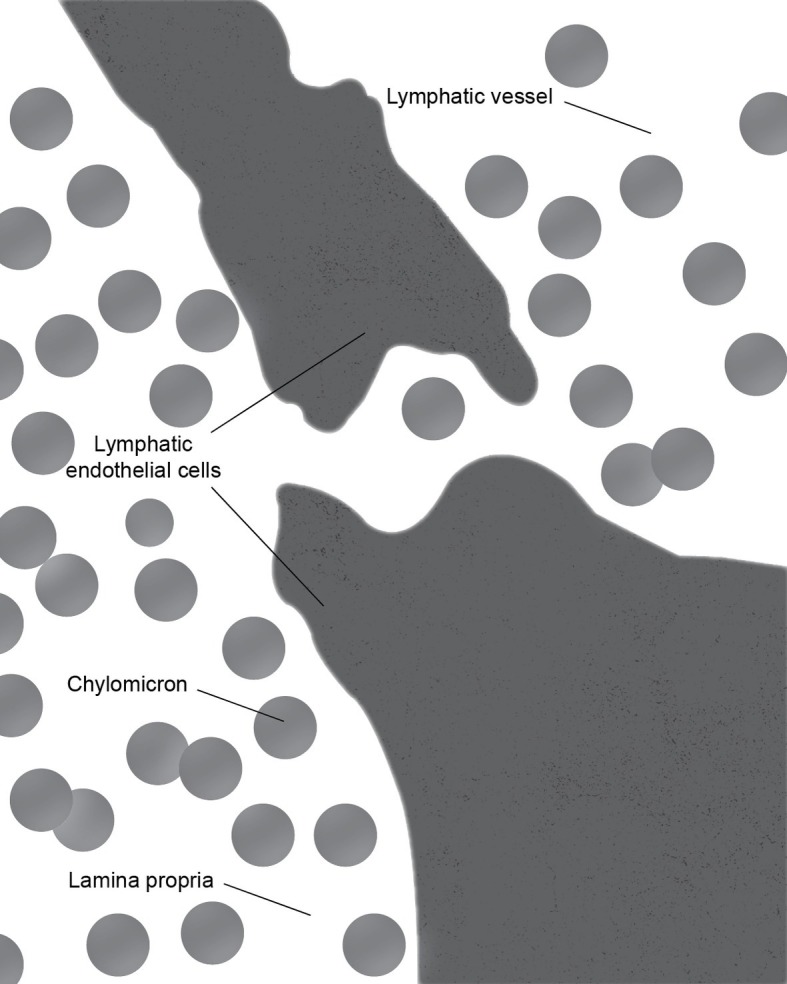
During the active lipid absorption, chylomicrons enter the lymph lacteal through a transcellular gap in the endothelial lining.

There is some controversy regarding the mechanism of chylomicron entry into the intestinal lacteals (Figure 3). The first two electron microscopic studies on the entry of fat as chylomicrons into the lacteals presented incompatible results. Palay and Karlin (1959) reported that chylomicrons passed through open intercellular junctions, while Ashworth et al. (1960) showed that they passed through “pores” in the lymphatic wall. A later study by Casley-Smith (1962) appeared to confirm the findings of Palay and Karlin. The study reported numerous electron microscopic images of open gaps between adjacent lymphatic endothelial cells containing chylomicrons, but they also observed chylomicrons entering the external endothelial membranes of the lacteal. However, Casley-Smith noted that the intercellular junctions of the intestinal lymph lacteals usually lack adhesion plates and that it is more likely that materials pass through the open junctions than through the endothelial cells. Furthermore, he suggested that Ashworth et al. may have mistaken these open junctions for “pores” (Casley-Smith, 1962). Several later studies concurred with Casley-Smith’s original assumption that a paracellular route was the most likely path of chylomicrons into the lymph lacteals (Figure 4). However, none conclusively ruled out transcellular transport through the endothelial cells (Casley-Smith, 1964; Rubin, 1966; Tytgat et al., 1971).

These conclusions were challenged by Dobbins (Dobbins and Rollins, 1970; Dobbins, 1971). With over 500 images examined, they concluded that a majority of the intercellular junctions remained tightly closed. Furthermore, he demonstrated that chylomicron-containing vesicles occupied approximately 15% of the cytoplasm within the endothelial cells. The average size of the chylomicrons is in the range of 1,600 Å. Thus, Dobbins concluded that transcytosis must be the primary means chylomicron uptake into the lacteals. Controversy over two transport mechanisms has continued and evidence supporting both has been reported. The findings of multiple studies by Azzali supported Dobbins’s conclusion (Azzali, 1982, 1990; Azzali et al., 2002). Meanwhile, Collan and Kalima (1974) demonstrated that two types of junctions were present between the endothelial cells of the lacteals: complicated and simple valve-like. They noted that the complicated joint areas served as a means of fixing adjacent endothelial cells together, while simple joint areas had an additional function as valves through which materials could be transported into the lacteal (Collan and Kalima, 1974).

A more recent study by Choe et al. (2015) has provided further insight into the mechanism of lipid drainage through the lacteals. Their study suggests that the contractility of the intestinal lacteals plays an important role in the uptake of chylomicrons into the lymphatic system. Furthermore, their findings suggest that rather than serving as passive conduits for lipids, the lacteals function as active pumps for lipid transport. Previous studies of villus motility in a dog model have described piston-like retractions and extensions. These motions were speculated to be mediated by the smooth muscles longitudinally aligned along the lacteal within in the intestinal villus (Womack et al., 1987, 1988). However, Choe and colleagues have demonstrated that the lacteals in a mouse model also undergo lateral contraction. In addition, they reported that interactions between the lacteals and surrounding smooth muscle resulted in enhanced absorptive ability and spontaneous contractibility that is regulated by the autonomic nervous system. They demonstrated that administration of acetylcholine and norepinephrine resulted in an increase and decrease in lateral contraction, respectively. These observations suggest that through autonomic regulation, the lymph lacteals serve a role in lipid transport as active pumps, which may also provide a critical mechanistic explanation for how the lacteals are able to drain such large quantities of dietary lipids. It should be emphasized that the role of the lacteals as pumps still need to be carefully studied and the same scenario may not apply to all animal species.

## Activation of Mucosal Mast Cells By Fat Absorption and the Metabolic Consequences

In addition to its role in the digestion and absorption of nutrients (e.g., fat), the gut also serves an important function in host defense. The gastrointestinal tract, a tube-like structure that is covered by mucosa from the oral cavity to the anus, is continuous with the external environment. Therefore, the gastrointestinal tract is a major entry site for many bacterial and viral pathogens and has a vastly diverse microbial community (Guarner and Malagelada, 2003; Palmer et al., 2007). Despite its constant and direct exposure to large quantities of microorganisms as well as foreign and dietary antigens, the gut mucosa maintains intestinal homeostasis by utilizing the mucosal immune system (Kagnoff, 2014; Mowat and Agace, 2014). Furthermore, the mucosal surfaces in the small intestine are protected from unfavorable antigen entry into the body and pathogen invasion through non-specific and specific immunological defense mechanisms (Turner, 2009). Non-specific mechanisms include a mucus layer and antimicrobial peptides (e.g., defensins and lysozymes) covering intestinal epithelial cells that provide a first line of defense against pathogen invasion (Johansson et al., 2008; Tokuhara et al., 2019). As an example of a specific mechanism, in response to luminal stimuli, intestinal epithelial cells produce pro-inflammatory chemokines and cytokines and induce the recruitment of immune cells in the lamina propria to the inflamed regions (Tokuhara et al., 2019).

Previous studies have suggested a close link between these intestinal immune cells and dietary fat absorption (Miura et al., 1998; Hara et al., 2003; Tsuzuki et al., 2006; Fujiyama et al., 2007; Ji et al., 2012). In particular, Ji et al. demonstrated that intestinal mucosal mast cells (MMC) in the lamina propria, key components of the mucosal immune system, are activated during fat absorption to release both pre-formed and *de novo* synthesized mediators in a conscious lymph fistula rat model (Ji et al., 2012). They found that intestinal MMC activation is dependent upon the quantity and type of fat infused intraduodenally and that long-chain fatty acids (LCFA) but not medium-chain fatty acids (MCFA) are effective in activating MMC. It is tempting to speculate that this observation is likely a result of the intestine’s handling of fatty acids (Sallee and Dietschy, 1973). MCFAs and short-chain fatty acids (SCFA), due to a relatively higher aqueous solubility than LCFA, are less dependent on luminal micellar solubilization for rapid uptake into enterocytes (Sallee and Dietschy, 1973). Furthermore, MCFAs and SCFAs are preferentially transported in the unesterified form bound to albumin *via* the hepatic portal vein, while LCFAs are preferentially packaged into chylomicrons in the enterocytes and transported through the lymphatic system (Ashbrook et al., 1972; Yang and Kuksis, 1987).

The difference in the intestinal handling of LCFAs and MCFAs and their differential effect on MMC activation suggest that the formation and secretion of chylomicrons is potentially linked to MMC activation. To test this possibility, Ji et al. administered L-81, an inhibitor of chylomicron formation. As expected, lymphatic transport of chylomicrons was completely abolished when L-81 was present in the intestinal lumen. However, eliminating the formation and transport of chylomicrons did not completely abolish the release of mediators by MMC (Ji et al., 2012). It is important to note that L-81 does not inhibit intestinal chylomicron formation instantly. It is possible that once chylomicron formation begins, the activation of MMC starts immediately. It is also entirely possible that there are other factors in addition to chylomicron formation and transport that are responsible for MMC activation by absorption of LCFA. As demonstrated by Chatterjee and Gashev, MMC are distributed in the intestinal lamina propria and are not directly exposed to intraluminal fatty acids (Chatterjee and Gashev, 2012). Thus, it is likely that signals mediated by intestinal epithelial cells, rather than direct interactions with lipid molecules, induce their activation (Ji et al., 2012).

Although results from Ji et al. demonstrated that fat absorption stimulates MMC activation, evidence from previous studies suggest that MMC activation is also important in enhancing the absorption and transport of lipids. It has been reported that intestinal permeability is increased during fat absorption, which would facilitate uptake of fluid and electrolytes (Kvietys et al., 1991). This could be explained by the finding that mediators released by activated mast cells affect intestinal paracellular permeability (Keita and Soderholm, 2010). Furthermore, *in vitro* studies have shown that mast cell mediator RMCPII directly increases epithelial permeability by decreasing the expression of the tight junction-associated proteins occludin and zonula occludens-1 (Scudamore et al., 1998). It has also been demonstrated that RMCPII can selectively attack collagen IV, which is a key component of intestinal basement membranes (Patrick et al., 1988). Thus, MMC activation may also contribute to the formation of the breakages in the basement membrane mentioned previously that would facilitate the transport of chylomicrons from intercellular space to the lamina propria. Together, these observations suggest that MMC activation may be involved in increasing intestinal permeability and creating discontinuities in the intestinal basement membrane that may facilitate the uptake and transport of fat (Ji et al., 2012). The underlying mechanisms linking fat absorption and MMC activation are not well understood and further studies are warranted. With a better understanding of these complex interactions, we may be able to modify the leaky gut phenomenon often associated with the consumption of a high fat diet.

## Link Between Chylomicron Transport, Mucosal Mast Cell Activation, Leaky Gut, and the Microbiome

In conscious lymph fistula rats, we demonstrated that treating the animals with antibiotics greatly suppressed the lymphatic transport of chylomicrons as well as the activation of mucosal mast cells normally associated with fat absorption (Sato et al., 2016). The antibiotics treatment resulted in very low bacterial counts that we were unable to get a taxonomic characterization of the remaining microbiome. The reduction in mucosal mast cell activation was determined by the reduction in the release of mucosal RMCPII into the lymph. In addition, we observed that antibiotics treatment reduced the intestinal release of diamine oxidase into the lymph as well as gut permeability, both of which normally increased dramatically as a result of active fat absorption. We found that the decrease in lymphatic lipid transport was also associated with a marked reduction in intestinal apolipoprotein B (apoB) secretion. Because ApoB is critical for the formation and secretion of intestinal chylomicron particles, it is reasonable to conclude that the reduction in apoB secretion may be responsible for the decreased lymphatic lipid transport. The intestinal secretions of ApoA-I and ApoA-IV were also reduced significantly. As far as we know, this is the first time it has been demonstrated that there is a link between intestinal chylomicron transport, mucosal mast cell activation, and the gut microbiota. More experiments are obviously needed to help us understand better the relation between them.

It is not clear how antibiotic treatment affects apolipoprotein output in enterocytes because our dose of penicillin and streptomycin is not known to affect mammalian cells. The question of antibiotics treatment on the transcriptional and the translational production and secretion of apolipoproteins is probably best studied *in vitro*, as there are less variables to content with than in the *in vivo* scenario. Of course, we cannot rule out in our experiment whether there is a direct effect of microbiome on apolipoprotein synthesis and secretion. In our study (Sato et al., 2016), when we allowed the restoration of microbiome in the antibiotics treated animals, there was also a recovery of intestinal lymphatic transport of lipids and apolipoproteins. These are very interesting questions that should be explored in future studies.

## Author Contributions

PT provided guidance on the overall direction of the manuscript. AZ and PT wrote the manuscript. JQ, ML, and PT edited and proofread the manuscript. All authors critically reviewed the final version of the paper.

### Conflict of Interest

The authors declare that the research was conducted in the absence of any commercial or financial relationships that could be construed as a potential conflict of interest.
